# Imaging of angiogenesis of human umbilical vein endothelial cells by uptake of exosomes secreted from hepatocellular carcinoma cells

**DOI:** 10.1038/s41598-018-24563-0

**Published:** 2018-04-30

**Authors:** Hiroshi Yukawa, Kaoru Suzuki, Keita Aoki, Tomoko Arimoto, Takao Yasui, Noritada Kaji, Tetsuya Ishikawa, Takahiro Ochiya, Yoshinobu Baba

**Affiliations:** 1 Department of Biomolecular Engineering, Graduate School of Engineering, Nagoya University, Furo-cho, Chikusa-ku, Nagoya, 464-8603 Japan; 2 ImPACT Research Center for Advanced Nanobiodevices, Nagoya University, Furo-cho, Chikusa-ku, Nagoya, 464-8603 Japan; 3 JST, PRESTO, Furo-cho, Chikusa-ku, Nagoya, 464-8603 Japan; 4 Department of Medical Technology, Nagoya University Graduate School of Medicine, Daikominami, Higashi-ku, Nagoya 461-8673 Japan; 5 Division of Molecular and Cellular Medicine, National Cancer Center Research Institute, 5-1-1 Tsukiji, Chuo-ku, Tokyo, 104-0045 Japan; 6 Institute of Innovation for Future Society, Nagoya University, Furo-cho, Chikusa-ku, Nagoya 464-8603 Japan; 7 Health Research Institute, National Institute of Advanced Industrial Science and Technology (AIST), 2217-14, Hayashi-cho, Takamatsu 761-0395 Japan; 8 College of Pharmacy, Kaohsiung Medical University, 100, Shin-Chuan 1 st Rd., Kaohsiung, 807 Taiwan R.O.C.

## Abstract

Hepatocellular carcinoma (HCC) is a typical hyper-vascular tumor, so the understanding the mechanisms of angiogenesis in HCC is very important for its treatment. However, the influence of the exosomes secreted from HCC cells (HCC-exosomes) on angiogenesis remains poorly understood. We herein examined the effects of the exosomes secreted from HepG2 cells (HepG2-exosomes) on the lumen formation of human umbilical vein endothelial cells (HUVECs) by the imaging of angiogenesis. The degree of lumen formation of HUVECs was dependent on the number of HepG2-exosomes. The HepG2-exosomes expressed NKG2D, an activating receptor for immune cells, and HSP70, a stress-induced heat shock protein associated with angiogenesis through the vascular endothelial growth factor (VEGF) receptor. In addition, the HepG2-exosomes contained several microRNAs (miRNAs) reported to exist in the serum of HCC patients. These results suggest that the HCC-exosomes play an important role in angiogenesis. Further studies on the function of HCC-exosomes may provide a new target for HCC treatment.

## Introduction

The incidence of human hepatocellular carcinoma (HCC) is rapidly increasing worldwide^1,2^. It is currently the third leading cause of cancer-related death with an annual incidence of over half a million cases, and the mortality rate is still high despite the improvements in the diagnostic and surgical techniques, and in perioperative care^3^. Currently, surgical techniques, including surgical resection, liver transplantation and ablative therapy, are regarded as the most efficient treatment methods^4,5^. However, the outcomes of treatment are not satisfactory^6^. In addition, the common methods used to treat various other cancers, such as radiotherapy and chemotherapy, are often not used for the treatment of HCC because of their toxicity and the insensitivity of the tumors to these agents^7^. Thus, a novel strategy for the treatment of HCC is needed.

HCC is a typical hypervascular tumor, so anti-angiogenic treatments have been receiving a lot of attention. In fact, the development and clinical trials of new agents, including molecularly-targeted anti-angiogenesis drugs are in progress. However, to ensure the progress of these studies in HCC, a better understanding of the mechanisms of angiogenesis in HCC is needed, and new strategies for HCC treatment based on these findings are highly anticipated.

Recently, it has become evident that various kinds of cells, including epithelial cells^8^, neurons^9^, dendritic cells^10^, T cells^11^, B cells^12^ and cancer cells secret exosomes. Exosomes are biological nanoparticles 30–100 nm in size, which are formed by the inward budding of multivesicular bodies (MVBs), which are a component of the endocytosis pathway^13,14^. Exosomes contain mRNAs, microRNAs (miRNAs) and proteins that can be transferred to target cells, inducing epigenetic and other changes, and are constitutively generated and released into the surrounding extracellular matrix and circulation through the fusion of MVBs with the plasma membrane^15^. However, thus far there is no evidence of the active targeting of exosomes. The exosomes have been shown to participate in cell to cell communication such as morphogen and RNA transport between cells^16^. In particular, cancer cells were reported to secret a large number of exosomes^17,18^. The exosomes secreted by cancer cells can influence the invasion of cancer cells, the immunological responses by T-cells and NK cells to the tumor^19,20^, and can also affect the angiogenesis of the surrounding endothelial cells. In fact, the exosomes secreted by various cancers, including breast cancer^19,21,22^, prostate cancer^22,23^ and renal cancer^24^, have been reported to affect the immunological response, the metastasis and niche formation of cancer cells and the angiogenesis around the tumor^25^.

It has been reported that HCC cell lines, such as HepG2 and Hep3B cells, secret exosomes^3^, and these exosomes can influence the natural killer cell antitumor responses^1^. However, the influence of the exosomes secreted by HCC cells (HCC-exosomes) on angiogenesis remains poorly understood. In this study, we evaluated the effects of the exosomes secreted by HepG2 cells (as representative HCC cells) (HepG2-exosomes) on the angiogenesis of human umbilical vein endothelial cells (HUVECs) by the imaging of angiogenesis.

## Results

### Characterization of the exosomes secreted by HepG2 cells

**Figure 1:**
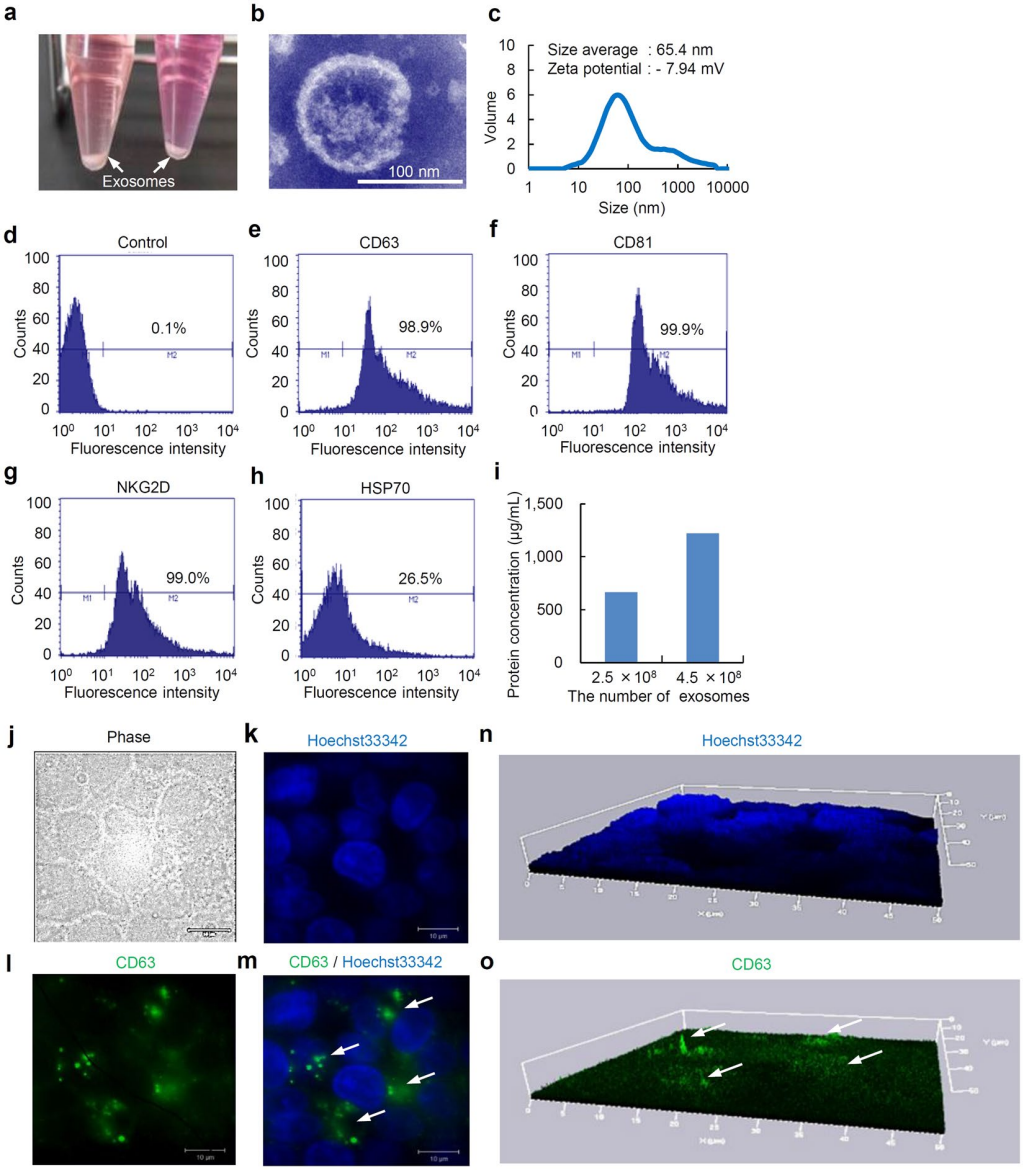
The isolation, characterization, and observation of exosomes secreted from HepG2 cells. (**a**) The exosomes secreted from HepG2 cells (HepG2-exosomes) isolated by the ExoQuick-TC kit in the bottom of the tubes were found as white pellets. Both white arrows show the exosomes. (**b**) An image of transmission electron microscopy of exosomes secreted from HepG2. (**c**) The size distribution, average size and zeta potential of HepG2-exosomes in distilled water. (**d–h**) The expression of marker molecules such as CD63, CD81, NKG2D and HSP70 on the surface of HepG2-exosomes. (**i**) The protein concentration of exosomes secreted from HepG2, as determined by the BCA method. (**j–m**) The exosomes in HepG2 cells were detected by using a FITC-labeled anti-human CD63 mouse monoclonal antibody. The morphology of the cells (**j**), nuclei labeled with Hoechst33342 (**k**), exosomes labeled with FITC-labeled anti-human CD63 mouse monoclonal antibody exist in HepG2 cells (**l**) and the merged images of the nuclei and exosomes (**m**) are shown. White arrows show the exosomes. (**n,o**) Three-dimensional images of nuclei labeled with Hoechst33342 (**n**) and exosomes bound to the FITC-labeled anti-human CD63 mouse monoclonal antibody (**o**) found in HepG2 cells. White arrows show the exosomes. These figures were obtained using superresolution structured illumination microscopy (SR-SIM, Carl Zeiss).

Exosomes collected from the supernatant of the culture medium in which HepG2 cells were incubated for four days were collected by the ExoQuick-TC kit in the bottom of a 15 mL tube (Fig. 1a). The exosomes were observed using a transmission electron microscope (TEM) (Fig. 1b). The average particle size was found to be 65.4 nm, ranging from 30 to 1000 nm, as determined by the zeta-size analysis. In addition, the zeta potential of the exosomes was −7.94 ± 0.83 mV (Fig. 1c).

Marker molecules expressed on the surface of exosomes could be detected when 4 µm beads were used as the size marker for the flow cytometric analysis. HepG2-exosomes expressed CD63 and CD81, which are known to be useful collection markers for exosomes (Fig. 1d–f). The expression of NKG2D, which is an activating receptor for NK, NKT, CD8 (+) and γδT cells, could also be detected (Fig. 1g). Moreover, about 25% of the exosomes expressed HSP70, a stress-induced heat shock protein (Fig. 1h). These data suggest that HepG2 cells produce exosomes, and that HepG2-exosomes could be successfully collected using our methods. The protein concentrations of the HepG2-exosomes (2.5 and 4.0 × 10^8^ particles) were 664.2 and 1220.8 µg/mL, and showed linearity with respect to the number of exosomes (Fig. 1i).

On the other hand, the exosomes present in HepG2 cells could be labeled by a FITC-labeled anti-human CD63 mouse monoclonal antibody and detected using superresolution structured illumination microscopy (Fig. 1j–o). White yarrows show the exosomes in HepG2 cells (Fig. 1m,o). These exosomes could be detected in the interspace between the nucleus and nucleus of HepG2 cells, thus indicating that the exosomes exist in the cytoplasm of HepG2 cells.

### Lumen formation of HUVECs activated by HepG2-exosomes

**Figure 2:**
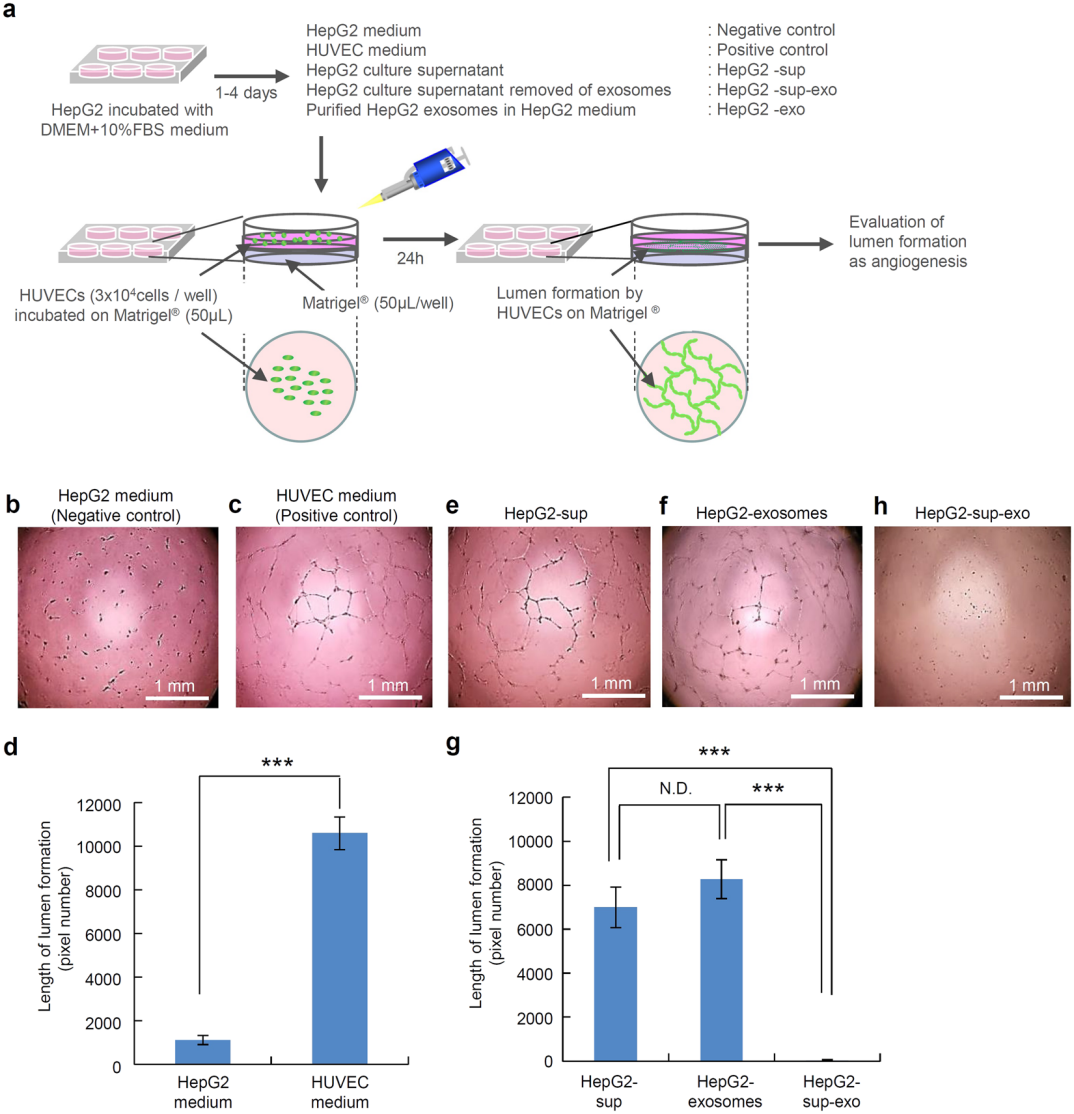
The lumen formation by HUVECs treated with HepG2-exosomes. (**a**) The schematic diagram of the experimental protocol is shown. The upper diagram shows the preparation of the culture medium and supplements, such as the HepG2 medium (negative control), HUVEC medium (positive control), HepG2 culture supernatant (HepG2-sup), purified HepG2 exosomes (HepG2-exosomes) and HepG2 culture supernatant with the exosomes removed (HepG2-sup-exo). The lower diagram shows the preparation used to assess the lumen formation of HUVECs in plates coated with growth factor-reduced Matrigel. The lumen formation of HUVECs on Matrigel was observed using a phase-contrast microscope. (**b,c,e,f,h**) The lumen formation by HUVECs under various culture conditions, such as HepG2 medium (negative control), HUVEC medium (positive control), HepG2 culture supernatant (HepG2-sup), purified HepG2 exosomes (HepG2-exosomes) added to HepG2 medium and HepG2 culture supernatant with the exosomes removed (HepG2-sup-exo). (**d**) The comparison of the length of the lumens formed by HUVECs between the HepG2 medium (negative control) and HUVEC medium (positive control). (**g**) The comparison of the length of the lumens formed by HUVECs between HepG2 culture supernatant (HepG2-sup), purified HepG2 exosomes (HepG2-exosomes) and HepG2 culture supernatant with the exosomes removed (HepG2-sup-exo). These data are shown as the means ± standard deviation of triplicate values. ***P < 0.001.

To investigate whether HUVECs are influenced by HepG2-exosomes and whether the exosomes induce angiogenesis, the HUVECs were cultured on Matrigel under various conditions (Fig. 2a). Lumen formation, as indicated by the presence of capillary-like structures, was found in the normal HUVECs medium (positive control), however, limited lumen formation was detected in the HUVECs cultured in HepG2 medium (negative control) (Fig. 2b,c). There was a significant difference between these two groups in terms of the length of the capillary-like structures determined by the pixel density (Fig. 2d). The HUVEC medium does not contain exosomes, whereas this medium contains the epidermal growth factor (EGF), fibroblast growth factor (FGF), vascular endothelial growth factor (VEGF) and insulin-like growth factor-1 (IGF-1) which induce the formation of HUVEC lumens. This data suggests that exosomes are not important when HUVECs are cultured in their preferred medium.

Of note, lumen formation could be induced by the HepG2 culture supernatant (HepG2-sup), in which HepG2 cells (1 × 10^5^ cells) had been cultured for four days, and in HepG2 medium supplemented with purified HepG2-exosomes (HepG2-exosomes) (Fig. 2e,f). There were no significant differences between these two conditions (Fig. 2g). On the other hand, when the exosomes were removed from at the HepG2 culture supernatant (HepG2-sup-exo), the lumen formation (capillary-like structures) could not be detected at all (Fig. 2h). There were significant differences between the HepG2-sup or HepG2-exosomes and HepG2-sup-exo in terms of the length of capillary-like structures (Fig. 2g). These data suggest that HepG2-exosomes can induce the lumen formation of HUVECs.

### Lumen formation of HUVECs that incorporated HepG2-exosomes

**Figure 3:**
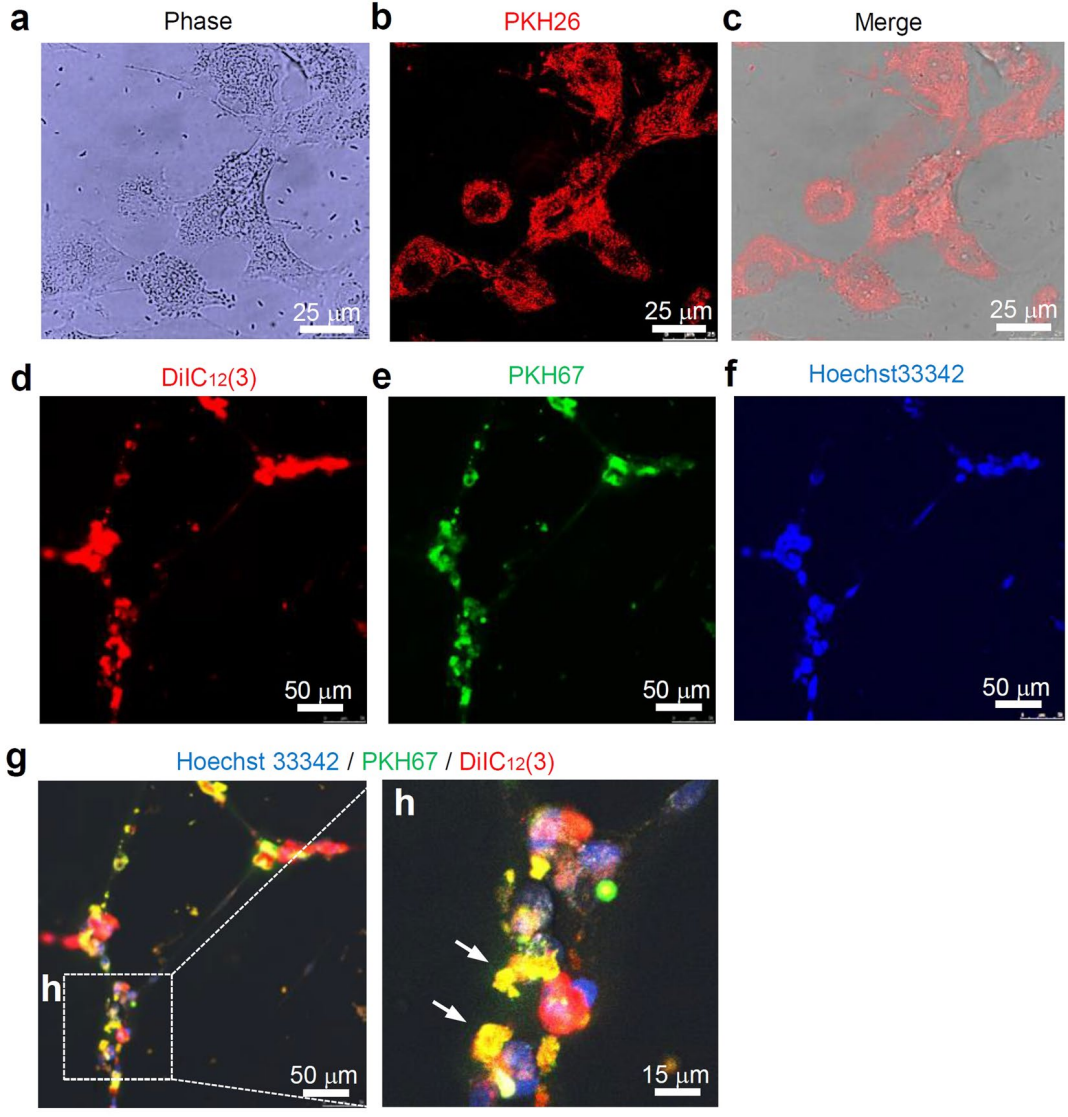
Angiogenesis imaging of HUVECs by the uptake of exosomes secreted from HepG2 cells. (**a–c**) The uptake of HepG2-exosomes labeled with PKH26 by HUVECs. These figures were imaged by confocal laser scanning microscopy. (**d–h**) The internalization of the HepG2-exosomes in the lumens formed by HUVECs. The membranes and nuclei of HUVECs were labeled with DilC_12_ (3) and Hoechst33342, respectively. HepG2-exosomes were labeled with PKH67. White arrows show the HepG2-exosomes included in the HUVECs that formed lumen structures. These figures were imaged by confocal laser scanning microscopy.

To investigate the uptake of HepG2-exosomes by HUVECs, HUVECs were incubated with PKH26-labeled exosomes. After 1 h, the red fluorescence derived from PKH26 was detected in the HUVECs by confocal laser scanning microscopy (Fig. 3a–c). In addition, to confirm that there was lumen formation by the HUVECs that had incorporated HepG2-exosomes, HUVECs labeled with DilC_12_ (3) and Hoechst33342 were allowed to incorporate the PKH67-labeled exosomes, and then were incubated for 24 h at 37 °C. The green fluorescence derived from the PKH67-labeled exosomes could be detected in HUVECs after the lumen formation (capillary-like structure). In the merged images, the orange or yellow fluorescence indicating the existence of exosomes could be detected in almost all HUVECs (Fig. 3d–h).

### Influence of the number and incubation time of HepG2 cells on the exosome production and the subsequent lumen formation by HUVECs

**Figure 4:**
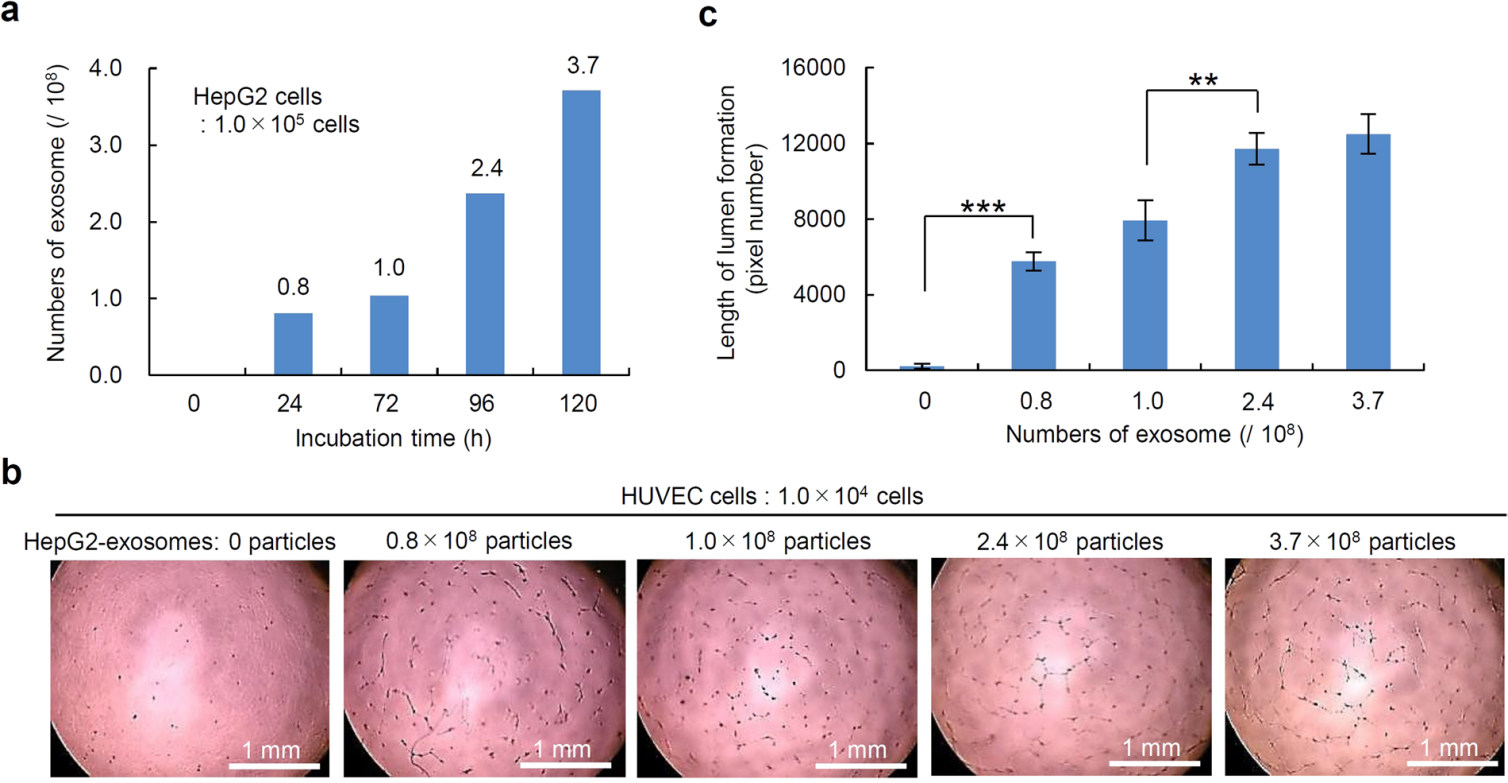
The influence of the length of incubation of HepG2 cells on the production of exosomes and the lumen formation by HUVECs. (**a**) The number of exosomes secreted from HepG2 cells (1 × 10^5^ cells) incubated for different lengths of time (0, 24, 72, 96 and 120 h) at 37 °C. (**b**) The lumen formation by HUVECs in the presence of exosomes secreted from HepG2 cells (1 × 10^5^ cells) incubated for different numbers of HepG2-exosomes. (**c**) The length of the lumen structures of HUVECs formed in the presence of exosomes secreted from HepG2 (1 × 10^5^ cells) incubated for different numbers of HepG2-exosomes. These data are shown as the means ± standard deviation of triplicate values. **P < 0.01, ***P < 0.001.

To check the influence of the incubation time of HepG2 cells on the exosome production, the number of exosomes in the culture media, in which HepG2 cells had been cultured for different times, was calculated by the ExoELISA kit. A total of 0.00, 0.81, 1.04, 2.37 and 3.71 × 10^8^ exosomes could be collected from the culture media, in which the HepG2 cells (1 × 10^5^ cells) had been incubated for 0, 24, 72, 96 and 120 hours, respectively (Fig. 4a). The influence of the number of HepG2-exosomes on the lumen formation of HUVECs was evaluated. The lumen formation (capillary-like structure) was observed to depend on the number of HepG2-exosomes (Fig. 4b,c). These results suggest that the number of exosomes increased with the increase in the number and incubation time of HepG2 cells, and the differences in the number of exosomes affected the degree of lumen formation of HUVECs.

### Detection of HepG2-exosomes and RNAs in the cytoplasm of HUVECs

**Figure 5:**
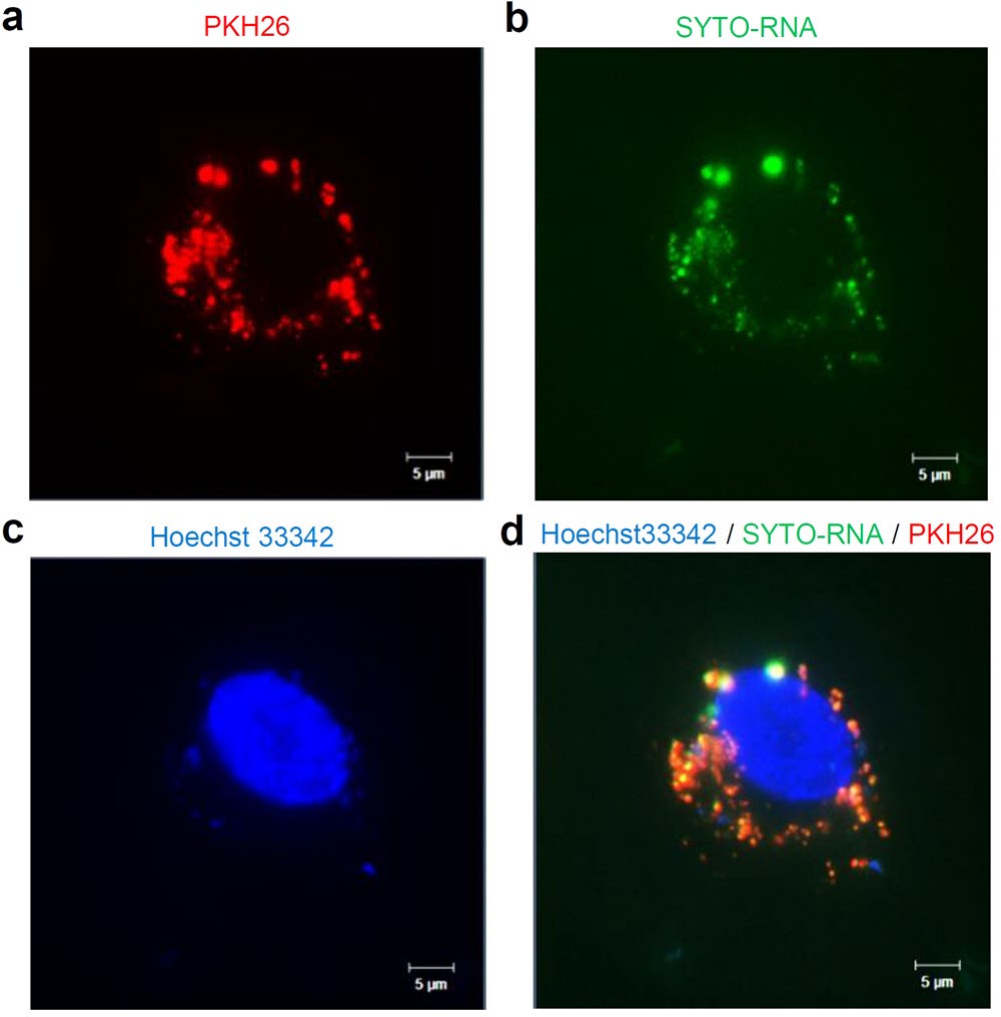
The labeling of HepG2-exosomes and RNAs in HUVECs. (**a**) The PKH26 labeled HepG2-exosomes incorporated in HUVECs. (**b**) The RNAs and miRNAs labeled with SYTO-RNASelect in HUVECs. (**c**) The nuclear of HUVECs labeled with Hoechst33342. (**d**) The merge image of HepG2-exosomes labeled with PKH26 and RNAs and miRNAs labeled with SYTO-RNASelect in HUVECs.

To detect the RNAs included in HepG2-exosomes, HUVECs were incubated with PKH26 labeled HepG2-exosomes. The PKH26 labeled HepG2-exosomes could be detected in HUVECs as red fluorescence in overnight incubation (Fig. 5a). Many RNAs in HUVECs treated with PKH26 labeled HepG2-exosomes could be detected by using SYTO-RNASelect as green fluorescence (Fig. 5b). The nucleus of HUVECs was labeled with Hoechst33342 (Fig. 5c). Both fluorescence of red and green derived from PKH26 and SYTO-RNASelect were observed to be matched with high probability (Fig. 5d). These data suggested that HepG2-exosomes incorporated in HUVECs include RNAs and miRNAs.

### miRNAs included in HepG2-exosomes

The miRNAs included in HepG2-exosomes were investigated using a miRNA array analysis. A list of miRNAs reported to exist in the serum of HCC patients is shown (Table 1). There were significant differences between the HCC and normal serum in the expression levels of these miRNAs. As a result, two differential miRNA expression patterns: sup- or down-regulated miRNAs, were identified in HCC serum. We examined whether the expression of these miRNAs could be detected in HepG2-exosomes, and showed the expression level based on the fluorescence intensity. All miRNAs listed in Table 1 could be detected in HepG2-exosomes (Table 2). Moreover, we investigated the top 20 up- or down-regulated miRNAs derived from HUVECs treated with HepG2-exosomes. The rank was based on the ratio of the fluorescence intensity of miRNAs derived from HUVECs treated with HepG2-exosomes to the fluorescence intensity of miRNAs derived from untreated HUVECs (Tables 3, 4). Some miRNAs such as miR-4456, miR-1285 and miR-6088, were expressed in normal HUVECs to a certain degree, however, the levels were remarkably reduced after the treatment with HepG2-exosomes (Table 3). Several up-regulated miRNAs were confirmed, and the levels of miRNAs such as miR-3186, miR-27, miR-421, miR-424, miR-377 and miR-362, were especially increased (Table 4), in spite of their lack of expression in HepG2-exosomes (data not shown). These results suggest that the level of miRNAs in HUVECs dramatically changed after the treatment with HepG2-exosomes.

**Table 1:** List of microRNAs associated with HCC patients.

MicroRNAs	Intensity of expression
miR-21	Increased expression in tumorous serum or liver tissues of HCC patients
miR-221, miR-224, miR-301	Increased expression in tumorous liver tissues compared with paired adjacent non-tumorous liver tissue from HCC patients or in tumorous serum
miR-222	Increased expression in tumorous liver tissues of HCC patients compared with paired adjacent non-tumorous or begin tumorous liver tissues
miR-122	Decreased expression in serum samples in HCC patients compared with normal subjects
miR-16, miR-195, miR-199a	Decreased expression in serum levels in HCC patients compared with normal subjects
miR-223	Decreased expression in tumorous liver tissues compared with paired adjacent non-tumorous liver tissues from HCC patients

**Table 2:** Intensity of microRNAs included in exosomes secreted from HepG2 cells.

MicroRNAs	Intensity of expression
has-miR-21–5p	51.4
has-miR-221–3p	14.2
has-miR-224-5p	10.0
has-miR-301b	6.4
has-miR-222-3p	10.8
has-miR-222-5p	5.1
has-miR-122-3p	4.9
has-miR-122-5p	35.6
has-miR-16-5p	42.5
has-miR-195-3p	18.2
has-miR-195-5p	4.4
has-miR-199a-5p	21.4
has-miR-199a-3p	5.2
has-miR-223-3p	28.8

**Table 3:** Top20 down-regulated microRNAs derived from HUVECs treated with exosomes of HepG2 cells.

Rank	miRNA	Intensity (HUVECs)	Intensity (HUVECs treated with exosomes of HepG2)	Ratio
1	has-miR-4456	56.4	5.2	0.09
2	has-miR-1285-5p	31.1	4.1	0.13
3	has-miR-4742-5p	13.7	3.3	0.24
4	has-miR-329	8.4	3.7	0.45
5	has-miR-4786-3p	9.4	4.5	0.48
6	has-miR-4776-3p	9.7	5.5	0.57
7	has-miR-3661	6.8	3.9	0.57
8	has-miR-491-3p	5.2	3.0	0.58
9	has-miR-4326	12.4	7.3	0.59
10	has-miR-4437	9.1	5.4	0.59
11	has-miR-5093	8.6	5.1	0.59
12	has-miR-4499	7.8	4.8	0.62
13	has-miR-4793-3p	8.6	5.4	0.62
14	has-miR-6088	3765.1	2339.9	0.62
15	has-miR-4512	6.1	3.8	0.63
16	has-miR-4418	8.2	5.2	0.63
17	has-miR-411-3p	11.5	7.7	0.67
18	has-miR-186-3p	9.4	6.5	0.69
19	has-miR-154-5p	6.9	4.8	0.69
20	has-miR-5589-5p	13.3	9.4	0.71

Ratio: Intensity of miRNA derived HUVECs treated with exosomes of HepG2/Intensity of miRNA derived from HUVECs.

**Table 4:** Top20 up-regulated microRNAs derived from HUVECs treated with exosomes of HepG2 cells.

Rank	miRNA	Intensity (HUVECs)	Intensity (HUVECs treated with exosomes of HepG2)	Ratio
1	has-miR-5088	73.0	432.8	5.93
2	has-miR-3186-5p	3.0	10.2	3.46
3	has-miR-3199	3.1	8.3	2.65
4	has-miR-145-5p	5.4	14.2	2.63
5	has-miR-3976	6.1	15.8	2.60
6	has-miR-27a-5p	3.3	8.3	2.55
7	has-miR-421	4.6	11.5	2.49
8	has-miR-5703	3.3	8.0	2.45
9	has-miR-424-5p	9.0	21.9	2.42
10	has-miR-765	13.5	31.7	2.34
11	has-miR-527	3.8	8.9	2.31
12	has-miR-518c-5p	4.3	9.8	2.30
13	has-miR-181b-3p	4.2	9.6	2.29
14	has-miR-513a-5p	58.7	133.7	2.28
15	has-miR-4709-3p	2.9	6.6	2.27
16	has-miR-432-3p	3.3	7.3	2.24
17	has-miR-377-3p	2.9	6.6	2.24
18	has-miR-362-5p	3.9	8.7	2.22
19	has-miR-218-5p	3.8	8.2	2.18
20	has-miR-3690	3.3	7.2	2.18

Ratio: Intensity of miRNA derived HUVECs treated with exosomes of HepG2/Intensity of miRNA derived from HUVECs.

## Discussion

It has recently been reported that exosomes secreted from cancer cells are involved in the malignant alternations of cells by the interaction with immune cells, such as T cells, B cells, macrophages and neutrophils, by the degradation of the extracellular matrix, by the induction of angiogenesis and by the niche formation, in which cancer stem cells exist^15,16,24,26–28^. Bobrie *et al*. showed that exosomes secreted from breast cancers accelerated metastasis by affecting the neutrophils^29^. Grange *et al*. showed that exosomes secreted from renal cancer cells have the potential to induce angiogenesis^24^. We confirmed that exosomes secreted from cervical cancer cells (HeLa cells) induced the lumen formation by HUVECs  (Supplementary Fig.).

The role of the exosomes derived from HCC cells is still being revealed. Li-Hong *et al*. showed that the exosomes secreted by HCC cells affect the antitumor responses of natural killer cells^1^. However, the effects of the exosomes secreted from HCC cells on angiogenesis are largely unknown. We herein investigated whether HepG2-exosomes have the ability to induce angiogenesis by evaluating the degree of lumen formation of HUVEC cells exposed to these exosomes.

We could observe the uptake of PKH26 or 67-labeled HepG2-exosomes by HUVECs within 1 h. The exosomes could be observed throughout the cytoplasm of HUVECs, and also entered the HUVECs nuclei after a longer incubation time. We also confirmed the lumen formation by HUVECs containing HepG2-exosomes. The production of exosomes was dependent on the number and the incubation time of HepG2 cells, and the degree of lumen formation was dependent on the number of HepG2-exosomes. Thus, these results suggest that HepG2-exosomes play important roles in angiogenesis. However, no significant difference in the length of lumen formation by HUVECs was observed between 2.4 × 10^8^ and 3.7 × 10^8^ HepG2-exosomes for 1.0 × 10^4^ HUVECs after 24 h of incubation. This result may indicate that the number of exosomes or length of lumen formation in this examination system had reached a maximum under these conditions. Therefore, no significant differences were detected following further increases in the number of exosomes or duration of the incubation.

It has been considered that some proteins and miRNAs involved in the expression of exosomal functions were included in exosomes. A previous study showed that HepG2-exosomes express tetraspanins such as CD9, CD63 and CD81 on their surface, and these results were similar to those of other types of exosomes^30^. The expression of NKG2D could also be detected on the surface of HepG2-exosomes. NKG2D is a known activating receptor for NK, NKT, CD8 (+) and γδT cells, suggesting that HepG2-exosomes influence the immune system^26,30^.

In addition, the expression of HSP70 could be detected on the surface of HepG2-exosomes in the present study. HSP70 also has reported to improve the tumor immunogenicity and induce a natural killer (NK) cell response^1^. Further, HSPs, including HSP70, have been reported to regulate VEGFR2 proteolysis, blood vessel development and repair^28^.Of note, it has been reported that HSP70 plays significant roles in endothelial cell migration and lumen formation via the phosphatidylinositol 3-kinase/Akt pathway^1,31^. Thus, the HSP70 expressed on the surface of HepG2-exosomes is considered to be one of the important molecules involved in the angiogenesis and immune response in HCC.

The intensities of many miRNAs in HepG2-exosomes were measured using a micro array analysis. The miRNAs previously reported to be associated with HCC, such as miR-16, -21, -122, -195, -199a, -221, -222, -223, -224 and -301, could all be detected in the HepG2-exosomes. Five of these miRNAs (miR-21, -221, -222, -224 and -301) have been reported to be upregulated in the tumor tissue of patients with malignant diseases, including HCC^32–37^. In particular, miR-21, -221 and -222 are overexpressed in many kinds of cancer. In contrast, other miRNAs have been reported to be down-regulated in the tumor tissue of patients with HCC^34,36–38^. In particular, mi-122 has been reported to have the ability to maintain the liver function, synthesize cholesterol, cause non-alcoholic steatohepatitis (NASH), induce the replication of hepatitis B virus and to induce liver cancer. At present, a locked nucleic acid (LNA) targeting miR-122 is being evaluated for the treatment of HCC in a phase II clinical trial sponsored by Santaris Pharma.

We also evaluated the up- or down-regulated miRNAs derived from HUVECs treated with HepG2-exosomes. The miRNAs on the list may induce the lumen formation by HUVECs. In fact, one of the downregulated miRNAs, miR-329, has previously been reported to suppress angiogenesis by targeting CD146. Endogenous miR-329 expression was downregulated by vascular endothelial growth factor and tumor necrosis factor-alpha, resulting in the elevation of CD146 in endothelial cells^39^. With regard to the up-regulated miRNAs, miR-145 has been reported to be associated with vascular smooth muscle cell proliferation, differentiation and phenotype switching^40^. The overexpression of miR-27 has been reported to significantly increase endothelial cell sprouting and angiogenesis by targeting semaphoring 6A^41,42^. The down-regulation of miR-424 has been reported to be associated with angiogenesis in non-small cell lung cancer. Hypoxia-induced miRNA-424 expression in human endothelial cells promotes angiogenesis^43,44^. Some of these miRNAs may therefore play important roles in the lumen formation by HUVECs. However, the main active molecules present in the exosomes leading to the lumen formation of HUVECs remain unclear. The identification of the main active molecules included in HepG2-exosomes and their mechanisms of action will provide insights into the angiogenesis caused by HepG2-exosomes.

## Methods

### Cells and materials

The stable human HCC cell line, HepG2, was purchased from the ATCC (Manassas, VA, USA). Normal human umbilical vein endothelial cells (HUVECs) were purchased from KURABO (Osaka, Japan). Dulbecco’s Modified Eagle Medium (DMEM), Endothelial Cell Basal Medium (EBM2), IDC Latex Particles and SYTO-RNASelects were purchased from Japan Life Technologies (Tokyo, Japan). ExoQuick-TC (Exosome Precipitation Solution) and ExoELISA (Exosome, CD9-ELISA Kit) were purchased from Systems Bioscience (California, America). The Micro BCA Protein Assay Kit was purchased from TAKARA BIO INC (Shiga, Japan). The miRNeasy Mini Kit was purchased from QIAGEN (Tokyo, Japan). Hoechst33342 was purchased from Wako Pure Chemical Industries Ltd. (Osaka, Japan). FITC-Anti-CD81 (Mouse-Mono (1D6)), FITC-Anti-CD63 (Mouse-Mono (MEM-259)) and FITC-Anti-CD314 (NKG2D) (Mouse-Mono (1D11)) were purchased from Japan Bio-Rad Laboratories, Inc (Tokyo, Japan). FITC-Anti-HSP70 (Mouse-Mono (C92F3A-5)) was purchased from StressMarq Biosciences Inc. (Victoria, Canada). DiIC_12_ (3), CSFE, BD Matrigel Matrix Growth Factor Reduced (GFR) and MICROTEST 96 were purchased from Japan Becton Dickinson and Company (Tokyo, Japan). PKH26 Red Fluorescence Cell Linker Kit and PKH67 Green Fluorescence Cell Linker Kit were purchased from Sigma Aldrich Japan (Tokyo, Japan).

### Cell culture

The HepG2 cells were cultured in DMEM supplemented with 10% fetal bovine serum (FBS) and 1% penicillin/streptomycin (HepG2 medium). HUVECs were cultured in EBM2 supplement with 2% FBS, hydrocortisone, EGF, FGF, VEGF, IGF-1, heparin, ascorbic acid, gentamicin and amphotericin B (HUVEC medium). Both cells were incubated under 5% CO_2_ at 37 °C.

### Isolation of exosomes

The commercially available ExoQuick-TC kit was employed as described by the vender for the isolation of HepG2-exosomes in the culture supernatant. Briefly, the culture medium secreted from HepG2 cells was centrifuged at 3,000 × g for 15 min to eliminate cells and cellular debris. The supernatant was filtered through a 0.45 µm filter (Millipore) in the vessel, and the same amount of ExoQuick-TC solution as the filtrate was added. The solution was incubated overnight at 4 °C, and then centrifuged at 1,500 × g for 30 min. After centrifugation, the exosomes appeared as a faint white pellet at the bottom of the vessel. The supernatant was removed, and then the exosomes could be obtained.

### Transmission electron microscopy

The samples were absorbed onto carbon-coated copper grids (400 mesh) and were stained with 2% phosphotungstic acid solution (pH 7.0) for 10 sec. The grids were then observed by a transmission electron microscope (JEM-1400 Plus, JEOL Ltd., Tokyo, Japan) at an acceleration voltage of 80 kV. Digital images (3296 × 2472 pixels) were taken with a CCD camera (EM-14830RUBY2, JEOL Ltd., Tokyo, Japan).

### Determination of the particle size distribution of exosomes

The particle size distribution, particle size average and zeta potential of exosomes in water were measured by a dynamic light-scattering spectrophotometer (ZETASIZER Nano-ZS, Malvern Instruments Limited).

### Quantification of exosomes

To quantify the amount of HepG2-exosomes, we assessed the amounts of CD9 expression and protein. The number of purified exosomes was measured by CD9-ExoELISA Kit. The protein content of purified exosomes was quantified by the BCA method. Both experiments were performed according to the manufacturer’s recommendations.

### Analysis of surface marker molecules of exosomes

The expression of marker molecules on the surface of exosomes was confirmed by a flow cytometric analysis on a FACS caliber (BD Biosciences) flow cytometer. Beads of IDC Latex Particles (4.0 µm) were used as a size marker for the flow cytometric analysis. Briefly, the IDC Latex Particles (5 µL, 1.4 × 10^7^ particles) and exosome solution (20 µg) in PBS (20 µL) were mixed with a rotor for 15 min at room temperature. Then, FITC-conjugated monoclonal antibodies (FITC-anti-CD63, -anti-CD81 -anti-CD314 (NKG2D) and -anti-HSP70) were added to the solution at the appropriate concentrations and incubated overnight at 4 °C. The solution was centrifuged at 3,000 rpm for 3 min, and the beads conjugated with exosomes were collected and washed with PBS supplemented with 2% FBS.

### Observation of exosomes present in HepG2 cells

To observe the presence of exosomes in HepG2 cells, HepG2 cells were incubated for at 37 °C on glass-bottomed 35 mm tissue culture dishes for 4 days, and the nuclei and exosomes were labeled with Hoechst33342 and a FITC-labeled anti-human CD63 mouse monoclonal antibody (FITC-Anti-CD63), respectively. After washing the samples with the culture medium three times, they were observed by superresolution structured illumination microscopy (SR-SIM (ELYRA), Carl Zeiss Co., Ltd.).

### Lumen formation assay

The formation of capillary-like structures (lumens) was assessed in 96-well plates coated with growth factor-reduced Matrigel. HUVECs (3 × 10^4^ cells/well) were seeded on top of the Matrigel (50 µL/well)-coated wells in each type of medium (HUVEC or HepG2 medium) and were treated with or without HepG2-exosomes. The organization on the Matrigel was recorded after 24 h at 37 °C using a phase-contrast microscope (Nikon ECLIPSE TS100). The total lumen area was quantified as the mean pixel density obtained from the image analysis of the microscopic field using the Image J software program. Briefly, the Image J software program was opened, then the file to measure the lumen length was selected. The “draw line” icon was selected and a line was traced around all of the lumen lines (area) of the HUVECs. Next, the “analyze” icon was selected and the length of all drawn lines was measured and summed. The data were expressed as the means ± SD of the tube length per field.

### Uptake of HepG2-exosomes by HUVECs

To detect the uptake of HepG2-exosomes by HUVECs, DilC_12_ (3)-labeled HUVECs were incubated for 1 h at 37 °C with PKH67 or PKH26-labeled HepG2-exosomes, and were washed with PBS, then were observed by fluorescence microscopy (OLYMPUS CKX41) or confocal microscopy (Leica TCS STED CW). To examine the lumen formation of the HUVECs that had incorporated exosomes, DilC_12_(3)-labeled HUVECs incorporating PKH67-labeled exosomes were incubated for 24 h at 37 °C on glass-bottomed 35 mm tissue culture dishes coated with growth factor-reduced Matrigel, and they were observed using confocal microscopy (Leica TCS STED CW). Hoechst 33342 was added for nuclear staining.

### MiRNA array analysis

The extraction of miRNA from HepG2-exosomes was performed using the miRNeasy Mini Kit according to the manufacturer’s instructions. Briefly, exosomes, including 700 µg proteins, were collected in a microtube, and 700 µL of QIAzol Lysis Reagent was added to each sample, which was then homogenized. The homogenate sample was incubated at room temperature (15–25 °C) for 5 min. Then, 140 µL of chloroform was added and the sample was shaken vigorously for 15 s, and incubated at room temperature for 2–3 min. The sample was centrifuged for 15 min at 12,000 × g at 4 °C, then was transferred to a new collection tube. Subsequently, 1.5 volumes (usually 525 µL) of 100 % ethanol were added, and the sample was mixed thoroughly by pipetting. Up to 700 µL of the sample, including any precipitate, was pipetted into an RNeasy Mini column in a 2 mL collection tube. The sample was centrifuged at >8000 × g for 15 s at room temperature, and the filtrate was discarded. The RNeasy Mini column was placed in a new 2 mL collection tube, and then was centrifuged at full speed for 1 min to further dry the membrane. Then, the RNeasy Mini column was transferred to a new 1.5 mL collection tube. The sample was reconstituted in 50 µL RNase-free water, and was centrifuged for 1 min at >8000 × g to elute the sample. The obtained miRNA was analyzed using a miRNA array tip (3D-Gene, TORAY). These data were obtained in cooperation with TORAY Industries, Inc.

### Statistical analysis

The statistical analysis was performed using the SPSS for windows software package, version 14.0. For the multiple group analyses, the homogeneity of the variance was assessed by the Leneve test. Parametric comparisons were examined using an analysis of variance (ANOVA). If the results of the ANOVA were significant, the significance of individual differences was evaluated using the Bonferroni test.

## Electronic supplementary material

Supplementary Information
