# Supplementary material for: Imaging of angiogenesis of human umbilical vein endothelial cells by uptake of exosomes secreted from hepatocellular carcinoma cells
Source: Sci Rep. 2018 Apr 30;8:6765. doi: 10.1038/s41598-018-24563-0 (PMC5928189; doi:10.1038/s41598-018-24563-0)
Supplement: Supplementary file 1 — Supplementary Information [file 41598_2018_24563_MOESM1_ESM.pdf]

Imaging of angiogenesis of human umbilical vein endothelial cells by uptake of exosomes secreted from hepatocellular carcinoma cells

Hiroshi Yukawa [1,2*], Kaoru Suzuki [1], Keita Aoki [1], Tomoko Arimoto [1], Takao Yasui [1,2,3], Noritada Kaji [1,2,3], Tetsuya Ishikawa [4], Takahiro Ochiya [5], Yoshinobu Baba [1,2,6,7,8*]

1) Nanobio Analytical Chemistry, Biomolecular Chemistry, Department of Biomolecular Engineering, Graduate School of Engineering, Nagoya University, Furo-cho, Chikusa-ku, Nagoya 464-8603, Japan
2) ImPACT Research Center for Advanced Nanobiodevices, Nagoya University, Furo-cho, Chikusa-ku, Nagoya 464-8603, Japan
3) JST, PRESTO, Furo-cho, Chikusa-ku, Nagoya 464-8603, Japan
4) Department of Medical Technology, Nagoya University Graduate School of Medicine, Daikominami, Higashi-ku, Nagoya 461-8673, Japan
5) Division of Molecular and Cellular Medicine, National Cancer Center Research Institute, 5-1-1 Tsukiji, Chuo-ku, Tokyo 104-0045, Japan
6) Institute of Innovation for Future Society, Nagoya University, Furo-cho, Chikusa-ku, Nagoya 464-8603, Japan
7) Health Research Institute, National Institute of Advanced Industrial Science and Technology (AIST), 2217-14, Hayashi-cho, Takamatsu 761-0395, Japan
8) College of Pharmacy, Kaohsiung Medical University, 100, Shin-Chuan 1 st Rd., Kaohsiung, 807, Taiwan, R.O.C.

***Corresponding authors:**
Hiroshi Yukawa, Ph.D., E-mail: h.yukawa@nanobio.nagoya-u.ac.jp
Yoshinobu Baba, Ph.D., E-mail: babaymtt@chembio.nagoya-u.ac.jp

Supplemental Figure

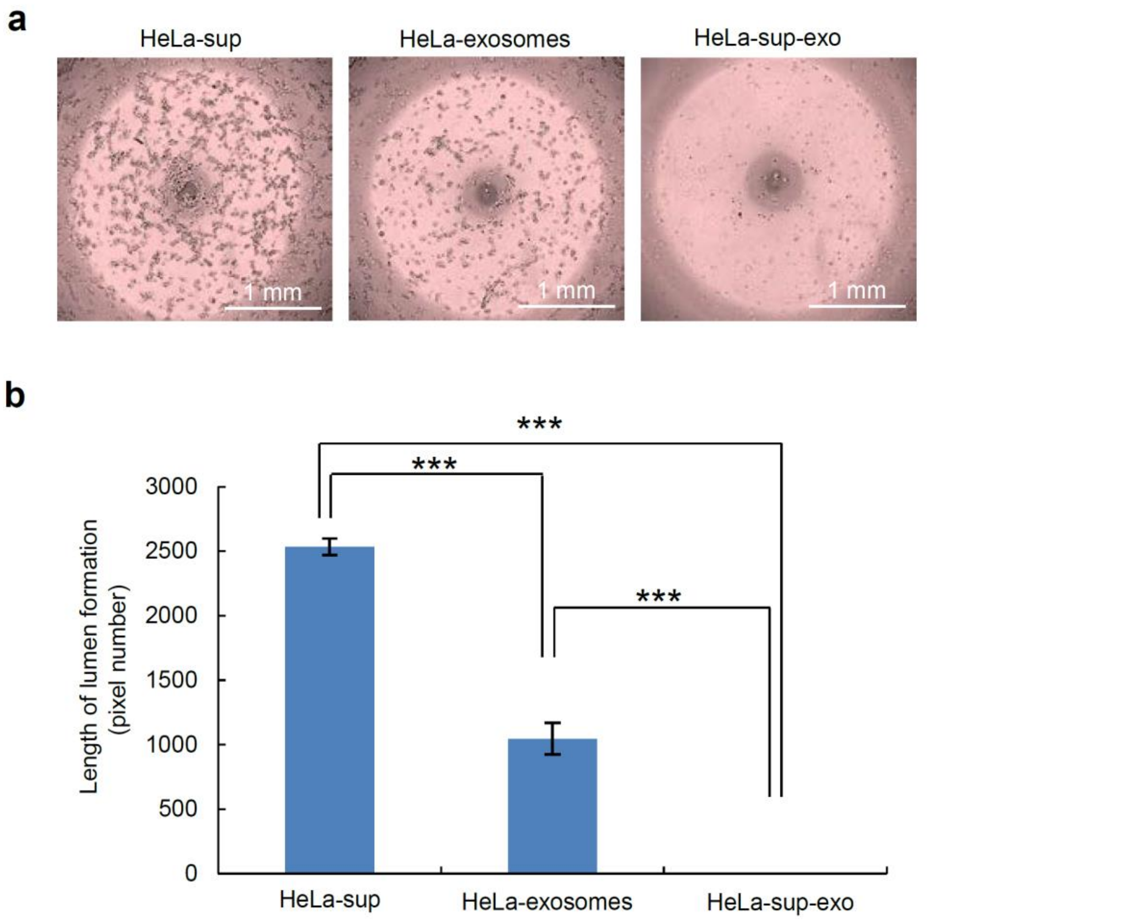

The lumen formation by HUVECs treated with exosomes secreted from HeLa cells.

(a) The lumen formation by HUVECs in the presence of exosomes secreted from HeLa cells (1×10^5 cells) incubated for different numbers of HepG2-exosomes. The lumen formation by HUVECs under various culture conditions, such as HeLa culture supernatant (HeLa-sup), purified HeLa exosomes (HeLa-exosomes) added to HeLa medium and HeLa culture supernatant with the exosomes removed (HeLa-sup-exo). (b) The comparison of the length of the lumens formed by HUVECs between HeLa culture supernatant (HeLa-sup), purified HeLa exosomes (HeLa-exosomes) and HeLa culture supernatant with the exosomes removed (HeLa-sup-exo). These data are shown as the means ± standard deviation of triplicate values. ***P<0.001.
